# Chronic androgen excess in female mice does not impact luteinizing hormone pulse frequency or putative GABAergic inputs to GnRH neurons

**DOI:** 10.1111/jne.13110

**Published:** 2022-03-10

**Authors:** Chris S. Coyle, Melanie Prescott, David J Handelsman, Kirsty A. Walters, Rebecca E. Campbell

**Affiliations:** ^1^ 2495 Centre for Neuroendocrinology and Department of Physiology School of Biomedical Sciences University of Otago Dunedin New Zealand; ^2^ Andrology Laboratory ANZAC Research Institute Concord Hospital University of Sydney Sydney NSW Australia; ^3^ 7800 Fertility and Research Centre School of Women’s and Children’s Health University of New South Wales Sydney NSW Australia

**Keywords:** androgens, GABA, GnRH, LH, membrane/nuclear, receptors

## Abstract

Polycystic ovary syndrome (PCOS) is associated with androgen excess and, frequently, hyperactive pulsatile luteinizing hormone (LH) secretion. Although the origins of PCOS are unclear, evidence from pre‐clinical models implicates androgen signalling in the brain in the development of PCOS pathophysiology. Chronic exposure of female mice to dihydrotestosterone (DHT) from 3 weeks of age drives both reproductive and metabolic impairments that are ameliorated by selective androgen receptor (AR) loss from the brain. This suggests centrally driven mechanisms in hyperandrogen‐mediated PCOS‐like pathophysiology that remain to be defined. Acute prenatal DHT exposure can also model the hyperandrogenism of PCOS, and this is accompanied by increased LH pulse frequency and increased GABAergic innervation of gonadotrophin‐releasing hormone (GnRH) neurons. We aimed to determine the impact of chronic exposure of female mice to DHT, which models the hyperandrogenism of PCOS, on pulsatile LH secretion and putative GABAergic input to GnRH neurons. To do this, GnRH‐green fluorescent protein (GFP) female mice received either DHT or blank capsules for 90 days from postnatal day 21 (*n* = 6 or 7 per group). Serial tail‐tip blood sampling was used to measure LH dynamics and perfusion‐fixed brains were collected and immunolabelled for vesicular GABA transporter (VGAT) to assess putative GABAergic terminals associated with GFP‐labelled GnRH neurons. As expected, chronic DHT resulted in acyclicity and significantly increased body weight. However, no differences in LH pulse frequency or the density of VGAT appositions to GnRH neurons were identified between ovary‐intact DHT‐treated females and controls. Chronic DHT exposure significantly increased the number of AR expressing cells in the hypothalamus, whereas oestrogen receptor α‐expressing neuron number was unchanged. Therefore, although chronic DHT exposure from 3 weeks of age increases AR expressing neurons in the brain, the GnRH neuronal network changes and hyperactive LH secretion associated with prenatal androgen excess are not evident. These findings suggest that unique central mechanisms are involved in the reproductive impairments driven by exposure to androgen excess at different developmental stages.

## Graphical abstract

Although chronic androgen exposure in female mice models the metabolic and reproductive impairments of polycystic ovary syndrome and up‐regulates androgen receptor expression in the hypothalamus, luteinizing hormone pulsatility is not elevated. Anatomical evidence for afferent inputs to gonadotrophin‐releasing hormone neurons, including spine density and vesicular GABA transporter appositions, also remain unchanged in adult female mice chronically exposed to androgen excess.

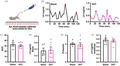

## 1 INTRODUCTION

Polycystic ovary syndrome (PCOS) is a common endocrinopathy, reported to affect approximately 10% of women of reproductive age around the world.^1^, ^2^ PCOS is characterised by the presence of at least two of three diagnostic criteria, including clinical and/or biochemical hyperandrogenism, oligo‐ or anovulation, and a polycystic morphology of the ovary.^3^ Approximately 75% of women diagnosed with PCOS present with luteinizing hormone (LH) hypersecretion,^4^, ^5^ and more than 90% of patients are likely to have an increased LH‐to‐follicle stimulating hormone ratio.^6^ PCOS is also associated with a number of co‐morbidities such as metabolic syndrome, which can include obesity, impaired glucose handling and insulin insensitivity.^7^ The aetiology for the diverse pathogenesis of PCOS is not clear and may be multifactorial.^8^

Although specific PCOS origins remain unclear, exposure to androgen excess is linked to the development of PCOS pathophysiology in women and in several pre‐clinical animal models.^9^ The key features of PCOS are recapitulated in non‐human primates,^10^ sheep,^11^ and rodents^12^, ^13^, ^14^ exposed to androgen excess. Of interest, different exposure paradigms drive the development of different PCOS‐like phenotypes. In the mouse, although chronic androgen exposure from 3 weeks drives anovulation and a metabolic syndrome,^13^, ^15^ acute prenatal exposure to androgen excess drives a lean PCOS‐like phenotype of anovulation and hyperandrogenism and that lacks a robust metabolic phenotype.^12^, ^14^ This suggests that exposure to androgen excess within different developmental windows contributes to the development of different disease phenotypes.

Irrespective of treatment paradigm, there is evidence across several pre‐clinical models suggesting that androgen excess‐mediated development of PCOS features is likely to involve the brain.^16^, ^17^, ^18^, ^19^ Many of the PCOS‐like traits observed following chronic exposure to the non‐aromatizable androgen dihydrotestosterone (DHT) from postnatal day (PND)21, including anovulation, increased body weight, adiposity, and dyslipidaemia, can be rescued by neuron‐specific deletion of the androgen receptor (AR).^15^ This suggests that a brain‐specific AR‐dependent mechanism mediates the impact of chronic DHT exposure on the development of acyclicity and metabolic features.^15^ However, the specific mechanisms remain to be determined. Acute, prenatal androgen (PNA) exposure results in increased gonadotrophin‐releasing hormone (GnRH) neuron spine density^20^ and increased GABA connectivity with GnRH neurons in both mice and sheep.^14^, ^20^, ^21^ In the PNA mouse, this reprogramming is associated with impaired negative feedback regulation of GnRH/LH secretion,^20^, ^22^ increased GnRH neuron firing,^14^, ^23^ and hyperactive LH secretion.^20^ The impact of androgen excess from a later developmental timepoint on these features remains to be determined in this common mouse model of PCOS‐like hyperandrogenism. Although female hyperandrogenism is associated with blunting negative feedback and a coincident rise in LH,^12^, ^24^ female androgen excess delivered in adulthood has been shown to blunt LH secretion in other models.^25^

Here, we used transgenic GnRH‐green fluorescent protein (GFP) mice,^26^ treated with chronic DHT from PND21 to model the hyperandrogenism of PCOS that promotes reproductive impairments and a metabolic syndrome,^13^, ^15^, ^27^ to assess LH secretion dynamics and anatomical evidence for GABAergic innervation to GnRH neurons. We also assessed the number of AR and oestrogen receptor α (ERα) immunoreactive cells within specific hypothalamic nuclei associated with steroid hormone feedback.

## 2 MATERIALS AND METHODS

### 2.1 Animals

Female GnRH‐GFP mice were used for all experiments and were bred and housed at the University of Otago Biomedical Research Facility (Dunedin, New Zealand). All protocols and procedures were approved by the University of Otago Animal Ethics Committee (Dunedin, New Zealand) and performed in accordance with the regulations of the Australasian and New Zealand Council for the Care of Animals in Research and Teaching. All animals were housed under a 12:12 h light/dark photocycle (lights on 6.00 AM) with food and water available ad libitum.

**FIGURE 1:**
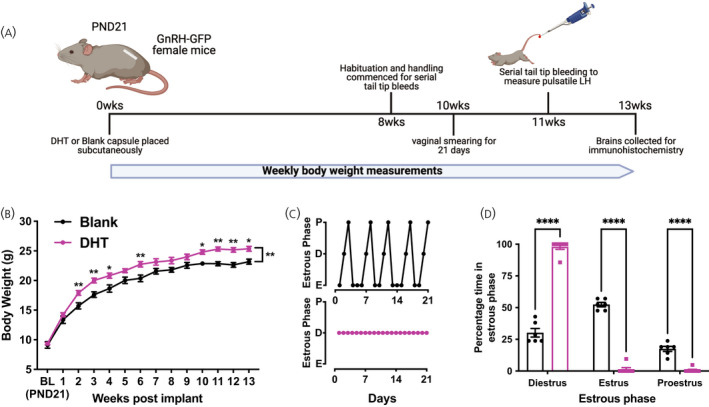
Chronic androgen excess causes an increase in body weight and loss of oestrous cyclicity. (A) Mean ± SEM weekly body weight (g) of animals with blank (*n* = 6) or dihydrotestosterone (DHT) (*n* = 7) capsules measured over 13 weeks. Asterisks above each weekly data point indicate the significance from Sidaks post‐hoc test; asterisks between lines indicate the mean effect of the capsule over 13 weeks (repeated measures two‐way ANOVA with Sidak's post‐hoc test). (B) Example oestrous cycle data collected over a 3‐week sampling window identifying when mice are in pro‐oestrus (P), dioestrus (D) or oestrus (E) for mice with Blank (B, black line) or DHT (B, magenta line) capsules. (C) Mean ± SEM percentage of time in each oestrous cycle phase. (D) Experimental timeline from capsule placement at postnatal day (PND)21. Schematic created using BioRender.com. Asterisks denote significant differences between treatment groups from two‐way ANOVA with Bonferroni's post‐hoc test. *p < .05, **p < .01, ****p < .0001

The hyperandrogenism of PCOS was modelled with chronic exposure to excess androgens from a peri‐pubertal timepoint.^13^, ^15^, ^27^ SILASTIC capsules (inner diameter, 1.47 mm; outer diameter, 1.95 mm; 1 cm; Dow Corning; 508–006) filled with approximately 10 mg of dihydrotestosterone (DHT) (*n* = 6) or left empty (blank; *n* = 7) were placed at PND 21 under isofluorane anaesthesia (2%). A small intrascapular incision was made and a s.c. pocket was created down the length of the back towards the tail with sterile forceps. The capsule was then placed into this pocket and the incision was sutured closed to keep the capsule in place for 90 days (13 weeks; experimental outline in Figure 1). Mice were weighed weekly, and daily handling and habituation training began from week 8 to prepare the mice for serial tail‐tip blood collection. Vaginal cytology was assessed from for the final 3 weeks of the experiment to assess oestrous cyclicity. At 13 weeks, when animals were in the dioestrous stage of the cycle, animals were killed humanely by pentobarbital overdose (3 mg per 100 μL), and then transcardially perfused with ice‐cold 4% paraformaldehyde (PFA) in 0.1 m phosphate buffer. Brains were dissected from the skull and post‐fixed for 1 h at room temperature (RT) in 4% PFA before being transferred to 30% sucrose in Tris‐buffered saline (TBS) overnight for cryopreservation. Once saturated, brains were sectioned coronally at a thickness of 30 μm on a freezing stage microtome (Leica 2400; Leica Biosystems) and collected in three series.

### 2.2 Measuring pulsatile LH

Mice were handled daily for habituation 4 weeks prior to serial tail‐tip blood collection. Sampling was carried out twice, 2 weeks apart on dioestrus. As previously described,^28^ 4 μL of whole blood was collected every 6 min for 2 h (10.00 AM to 12.00 PM) from a small incision in the tail‐tip. Blood was then immediately suspended in 50 μL of phosphate‐buffered saline with 0.05% Tween‐20 before being snap frozen on powdered dry ice. Samples were stored at −20°C until LH was measured by an ultrasensitive sandwich enzyme‐linked immunosorbent assay (ELISA), as described previously.^20^, ^28^, ^29^ Briefly, 96‐well high affinity binding plates were coated with 50 μL per well of bovine LHβ518B7 monoclonal antibody (dilution 1:1000 in PBS; RRID: AB_2756886; Pablo Ross, UC Davis, Davis, CA, USA) to serve as a capture antibody. A rabbit polyclonal antibody (dilution 1:10,000; AFP240590Rb; RRID: AB_2665533; National Hormone and Pituitary Program, Torrance, CA, USA) was used to detect bound LH in conjunction with a polyclonal‐horseradish peroxidase (‐HRP) conjugated goat anti‐rabbit secondary antibody (dilution 1:1000; RRID: AB_2617138; DAKO). LH concentration was determined using a mouse LH‐RP reference provided by Albert F. Parlow (National Hormone and Pituitary Program, Torrance, CA, USA). The sensitivity of this LH ELISA was 0.04 ng mL^–1^, and the intra‐ and inter‐assay coefficients of variance were 5.7% and 4.3%, respectively. LH pulses were detected using the recently reformulated PULSAR software with G values optimised for detecting LH pulses in intact female mice, G1 = 3.5, G2 = 2.6, G3 = 1.9, G4 = 1.5, and G5 = 1.2.^30^ LH pulse amplitude for each pulse was computed within PULSAR for each pulse and basal LH was calculated as the average of the 10 lowest values (that were not zero values). Data collected across the two sampling periods was averaged for each animal.

### 2.3 Immunohistochemistry

Sections containing the rostral preoptic area (rPOA) were selected from a single 1:3 series and washed thoroughly in TBS before staining for vesicular γ‐aminobutyric acid transporter (VGAT) and GFP (to visualise GnRH neurons). Free‐floating immunohistochemistry was performed as reported previously^20^, ^31^, ^32^ with primary antibody omission serving as a negative control. Briefly, sections were blocked with 2% normal goat serum (NGS) in TBS with 0.3% Triton X‐100 and 0.25% bovine serum albumin (BSA) for 30 min. Sections were then incubated with polyclonal rabbit anti‐VGAT primary antibody (dilution 1:750; RRID: AB_887869; Synaptic Systems) for 48 h at 4°C. VGAT staining was detected using goat anti‐rabbit AlexaFluor568 (dilution 1:500; RRID: AB_143157; Molecular Probes, Invitrogen) before subsequent incubation with polyclonal chicken anti‐GFP (dilution 1:5000; RRID: AB_2307313; Aves Labs Inc.) overnight at RT, and visualised with goat anti‐chicken AlexaFluor488 (dilution 1:500; RRID: AB_142924; Molecular Probes, Invitrogen).

To visualise AR and ERα expressing cells throughout the hypothalamus, a 1:3 series of brain sections containing the full hypothalamus from the POA through to the caudal extent of the arcuate nucleus (cARN) was labelled for each steroid hormone receptor. Free‐floating sections were washed thoroughly in TBS overnight at 4°C before chromogenic immunostaining. Sections for AR labelling were incubated in 0.01 m Tris‐HCL (pH 10.0) at 95°C for 10 min for antigen retrieval and then left at RT for 20 min to cool. Sections were then washed in TBS and transferred to 10% fetal bovine serum in TBS for 1 h before peroxidase quenching in TBS containing 40%v/v methanol and 3%v/v H_2_O_2_ for 30 min. Sections for ERα labelling only required peroxidase quenching before being incubated in primary anti‐sera. Sections were washed in TBS and then transferred to incubation solution (TBS with 0.3% Triton X‐100, 0.25% BSA, 2% NGS) containing either rabbit anti‐AR antibody (dilution 1:500; RRID: AB_310214; Merck‐Millipore; PG‐21, 06–680) or rabbit anti‐ERα antibody (dilution 1:10,000; RRID: AB_310305; Merck‐Millipore; 06–935) for 72 h at 4°C. Following TBS washes, tissue was placed in incubation solution containing a biotinylated goat anti‐rabbit IgG secondary (dilution 1:500: RRID: AB_2313606; Vector Labs; BA‐1000) for 90 min at RT. Immunolabelling was revealed using the ABC‐HRP Vectastain Kit (dilution 1:200, Vector Labs, PK‐4000) with glucose oxidase and nickel‐enhanced 3,3′‐diaminobenzidine to produce a black precipitate.

### 2.4 Microscopy and image analysis

GnRH neurons were imaged using an inverted Nikon A1R confocal microscope (Nikon Instruments Inc.), with 488 and 543 nm lasers. GnRH neurons (8–10 per animal) were randomly selected across two representative rPOA sections from each animal (DHT, *n* = 6; blank, *n* = 6) and images were captured at 40× magnification (0.5μm Z‐step, 1 AU pinhole, digital magnification 2×). Using NIS‐Elements AR 4.5 (Nikon Instruments Inc.), images were analysed for VGAT appositions and GnRH spines on the GnRH neuron soma and at intervals of 15 μm along the primary dendrite as previously described.^20^, ^31^, ^32^ VGAT puncta were considered in close apposition to GnRH neurons when there was an absence of black pixels between the cyan and magenta signals. Spines were defined as spiny protrusions from the cell body measuring greater than 1 μm and less than 5 μm.^33^, ^34^

AR and ERα stained brain sections were imaged using brightfield light microscopy on an Olympus BX‐51 microscope (Olympus Corporation). Sections through the rostral periventricular nucleus of the third ventricle (RP3V), including the anteroventral periventricular nucleus (AVPV) and the periventricular nucleus (PeN), were imaged using a 10× objective lens. Sections including the rostral, middle, and caudal levels of the arcuate nucleus (ARN) were imaged using a 20× objective lens with all images being captured on a Jentopix camera. The number of ERα positive cells was quantified in one or two representative sections containing each region (AVPV, PeN, rARN, mARN, and cARN) using ImageJ (NIH) and averaged for each region. The number of AR positive cells in each area was quantified in 1–2 representative sections containing each region using QuPath^35^ with thresholding parameters of 0.15 for RP3V images and 0.08 for ARN images. Positively labelled cells were defined within a region of interest (ROI) with defined borders for each nucleus. The ROI defined for each region was used consistently across animals.

Additionally, AR and ERα staining was semi‐quantitively assessed throughout the whole rostral to caudal extent of the hypothalamus using an Aperio SlideScanner with a 20× objective lens for two animals from each group. For semi‐quantative analysis of AR and ERα, − denotes an absence of staining, + denotes the presence of a few positively labelled cells, ++ denotes the presence of many positively stained cells with enough to clearly demarcate specific nuclei, and +++ denotes the robust expression of positively labelled cells.

### 2.5 Statistical analysis

All statistical analyses were carried out in Prism, version 9.0 (GraphPad Software Inc.). All data are presented as the mean ± SEM unless otherwise stated. Normality of the data was tested using the Shapiro–Wilk test to assess whether parametric or non‐parametric statistical tests should be performed. Body weight data was analysed by repeated measures two‐way ANOVA with Bonferroni's post‐hoc test for weekly comparisons between blank and DHT. Percentage of time spent in each oestrous cycle stage was analysed using two‐way ANOVA, with Bonferroni's post‐hoc test being used to compare between blank and DHT capsules for each phase of the cycle. Pulsatile LH characteristics were analysed using a Student’s *t* test, with the exception of pulse frequency, which was analysed by a Mann–Whitney *U* test. Total VGAT apposition density and GnRH spine density data were analysed by a Student's *t* test, with neuronal subcompartments including the soma and 15‐μm bins along the primary dendrite being analysed using a repeated measures two‐way ANOVA with a Bonferroni post‐hoc test. AR and ERα positive cell counts were analysed using an unpaired Student’s *t* test for each region analysed. *p* < 0.05 was considered statistically significant.

## 3 RESULTS

### 3.1 Recapitulation of the chronic androgen excess model

Mice with DHT capsules inserted at PND21 (Figure 1A) exhibited significant weight gain compared to blank capsule controls (*F*_1,11_ = 11.1; *p*_mean effect of treatment_ = .0067) (Figure 1B;) as observed previously.^13^ DHT treated mice also exhibited a pronounced loss of oestrous cyclicity (Figure 1C,D), with the majority of mice remaining completely acyclic. DHT treated mice spent significantly more time in dioestrus compared to controls (two‐way ANOVA; *F*_2,33_ = 403.8; *p*_mean effect of oestrous cycle_ < .0001) (Figure 1D) and a reduced amount of time in both oestrus and pro‐oestrus (*p* < .0001) (Figure 1D).

### 3.2 Chronic androgen excess does not alter LH pulsatility

**FIGURE 2:**
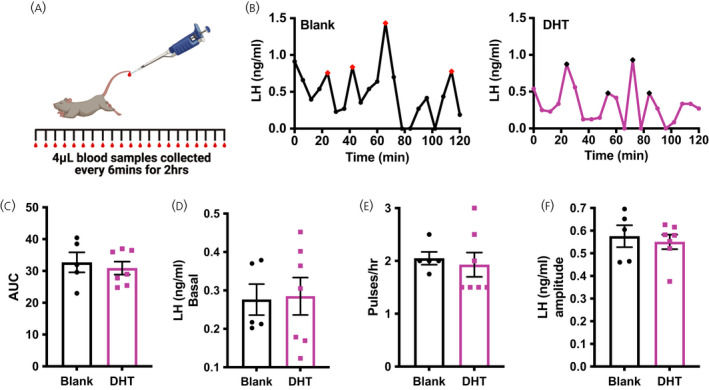
Chronic exposure to dihydrotestosterone (DHT) does not impact pulsatile lutenizing hormone (LH) secretion. (A) Diagram of tail‐tip bleeding protocol. (B) Representative patterns of LH secretion over 2 h from mice with either a blank (black) or DHT (magenta) capsule in dioestrus. Pulses indicated by a red or black data point, respectively. (C) Average LH area under the curve (AUC) over the 2‐h bleed. (D) Average basal LH concentration. (E) Average pulse frequency. (F) Average LH pulse amplitude for pulses identified by PULSAR. Schematic created using BioRender.com. Blank, *n* = 5 and DHT, *n* = 7 for all measurements; (C, D, F) Unpaired Student's *t* tests; (E) Mann–Whitney *U* test

LH secretion dynamics over time were measured with serial tail‐tip blood sampling from intact female animals in dioestrus (Figure 2A,B). The total amount of LH released over the 2‐h sampling window (calculated as area under the curve) was not different between blank and DHT‐treated mice (*t*_11_ = 0.4987; *p* = .6288) (Figure 2C). Likewise, there were no differences detected in the basal level of LH (*t*_11_ = 0.1297; *p* = .8994) (Figure 2D), LH pulse frequency (*U* = 12, *p* = .375) (Figure 2E) or the amplitude of LH pulses (*t*_11_ = 0.4512; *p* = .6608) (Figure 2F) between groups.

### 3.3 Chronic androgen excess does not affect GnRH neuron spine density or putative GABA appositions

**FIGURE 3:**
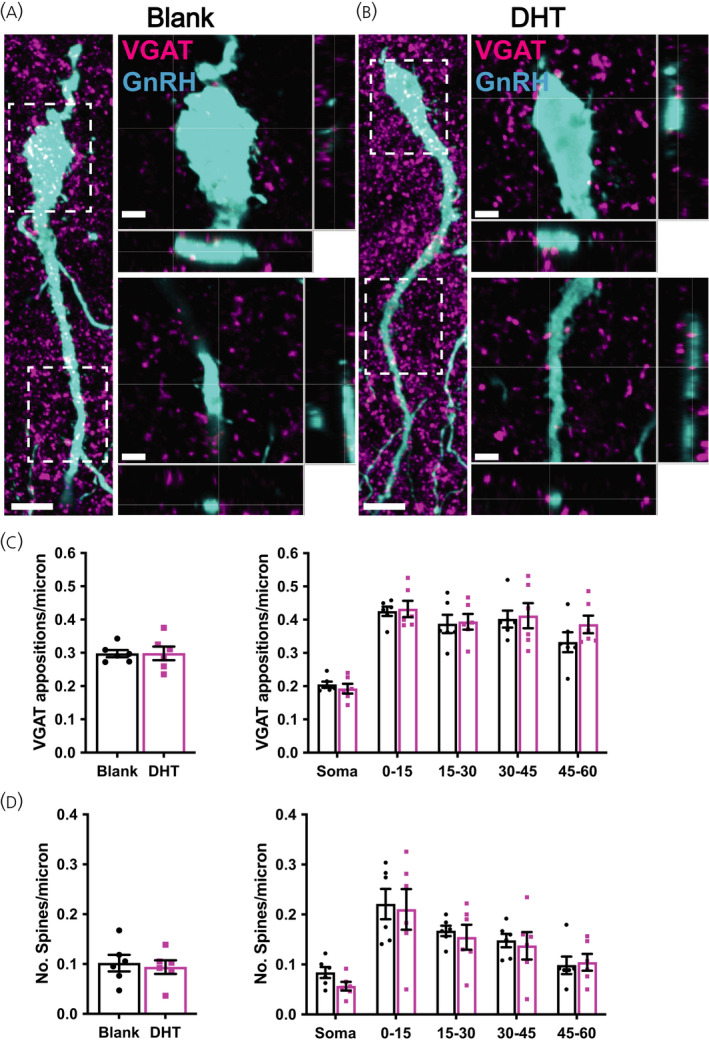
Chronic dihydrotestosterone (DHT) exposed females do not exhibit changes in gonadotrophin‐releasing hormone (GnRH) neuron spine density or GABAergic close appositions. (A, B) Representative confocal images of green fluorescent protein expressing GnRH neurons (cyan) and vesicular GABA transporter (VGAT) immunoreactive puncta (magenta) from animals with Blank (A) or DHT (B). Images depicting GnRH neuronal cell body and the proximal primary dendrite are collapsed maximum projection images; Scale bars =10 μm. Inset images of the soma and primary dendrite (framed in dotted line) are a single plane equating to a thickness of 0.5 μm; Scale bars =3 μm. Closed arrowheads denote some close VGAT immunoreactive appositions (magenta) onto the GnRH neuron; Open arrowheads denote some GnRH neuron spines. (C) Mean ± SEM number of VGAT immunoreactive appositions per micrometre of the total neuron, and in somatic and 15‐μm regions of the primary dendrite. Mean ± SEM number of spines per micrometre for the whole GnRH neuron and in somatic and 15 μm regions of the primary dendrite. *n* = 6 per group; 52–54 neurons per animal

To determine whether the chronic DHT model exhibits altered GnRH neuron morphology or evidence of enhanced GABAergic input, the number of VGAT puncta (magenta) in close apposition with the GnRH neuron soma and primary dendrite (cyan) was quantified (Figure 3A,B). Immunolabelling of GFP in brain tissue from GnRH‐GFP mice enabled robust visualisation of GnRH neuron morphology, including lengthy dendrites and spiny protrusions. Labelling of VGAT revealed widespread punctate labelling and VGAT puncta were frequently found closely apposed with the GnRH neuron soma and dendrite (Figure 3A,B, solid arrowheads). No differences in the total density of VGAT appositions to GnRH neurons were detected between DHT treated animals and blank controls (*t*_10_ = 0.02653; *p* = .9794) (Figure 3C), nor were any differences evident in any specific region of the neuron (repeated measures two‐way ANOVA; *F*_1,10_ = 0.2460, *p*_mean effect of treatment_ = .6306; *p* = .99 for all post‐hoc tests) (Figure 3C). Total GnRH neuron spine density was also found to be similar between DHT and blank animals (*t*_10_ = 0.3643; *p* = .7232) (Figure 3D). Likewise, the spine density in specific neuronal sub‐compartments, including the soma and 15μm intervals along the primary dendrite, was also not different between groups (repeated measures two‐way ANOVA; *F*_1,10_ = 0.1859; *p*_mean effect of treatment_ = .6755; *p* = .99 for all post‐hoc tests) (Figure 3D).

### 3.4 Chronic androgen excess increases AR but not ERα immunoreactivity in the hypothalamus

**FIGURE 4:**
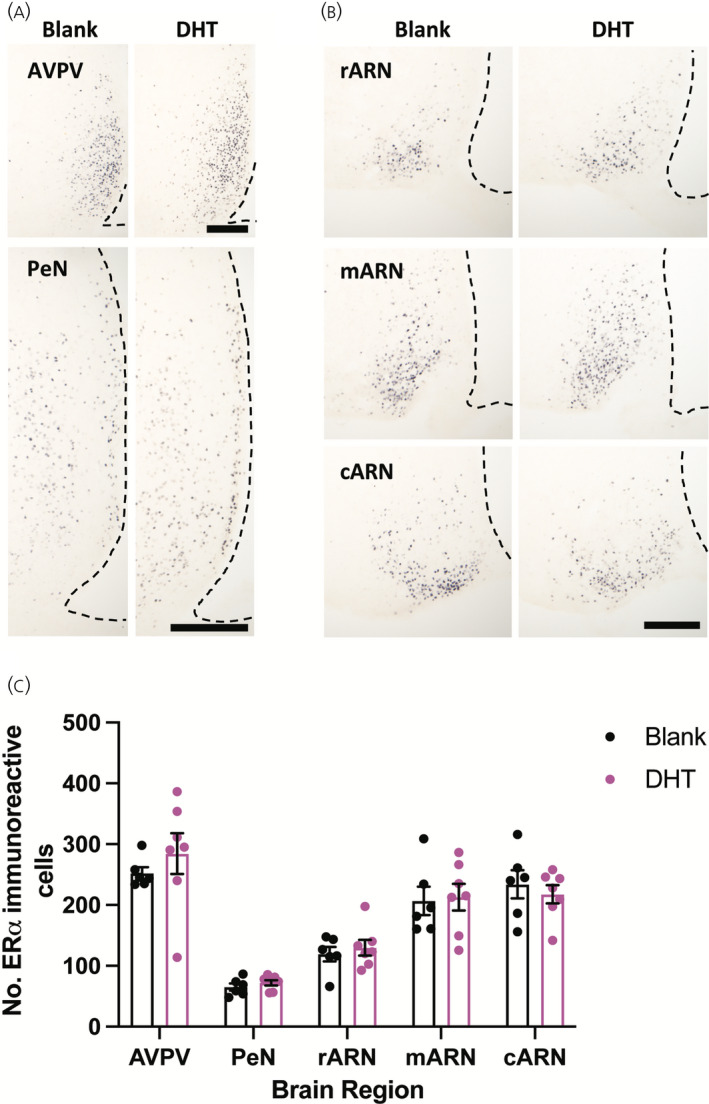
The number of oestrogen receptor α (ERα) expressing neurons in the hypothalamus is not changed following chronic dihydrotestosterone (DHT) exposure. (A) Representative images of ERα labelling in the rostral periventricular nucleus of the third ventricle, including the anteroventral periventricular nucleus (AVPV) and periventricular nucleus (PeN) in female mice treated for 3 months with either a blank or DHT containing capsule; Scale bars =200 μm. (B) Representative images of ERα labelling in the rostral arcuate nucleus (rARN), middle arcuate nucleus (mARN), and caudal arcuate nucleus (cARN) in females treated for 3 months with either a blank or DHT containing capsule; Scale bars =200 μm. (C) Mean ± SEM number of ERα immunoreactive cells quantified in each hypothalamic area. *n* = 6 or 7 per group, unpaired Students *t* test

**FIGURE 5:**
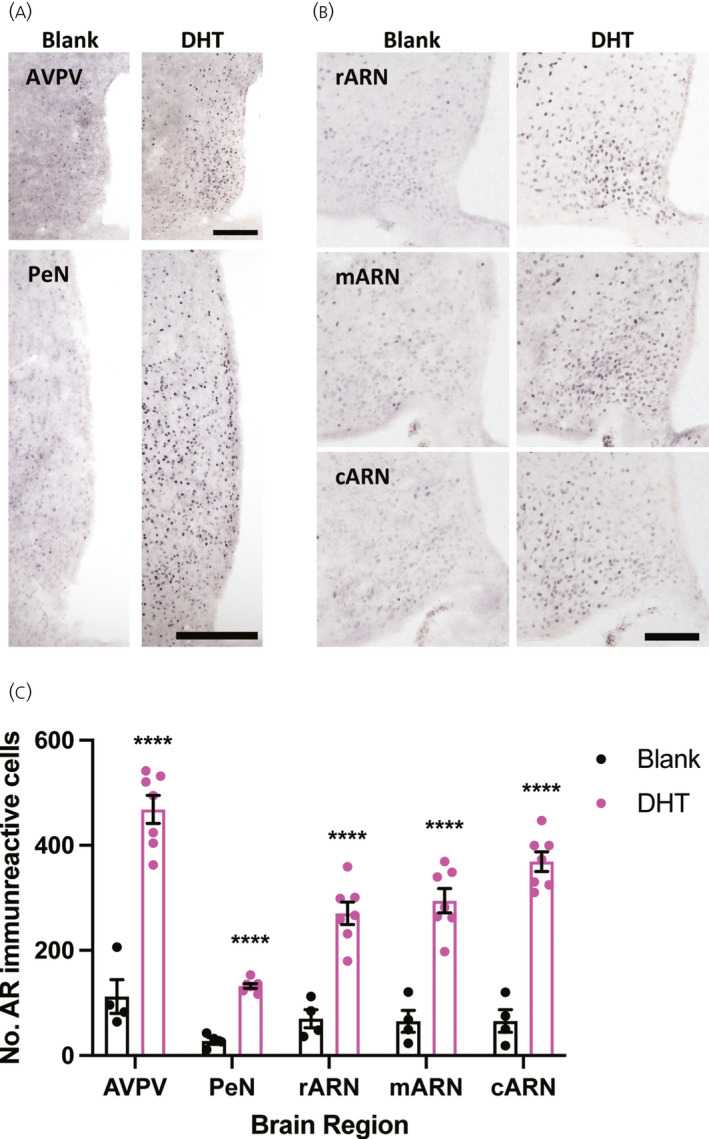
The number of androgen receptor (AR) expressing neurons is significantly increased in the female hypothalamus following chronic dihydrotestosterone (DHT) exposure. (A) Representative images of AR labelling in the rostral periventricular nucleus of the third ventricle, including the anteroventral periventricular nucleus (AVPV) and periventricular nucleus (PeN) in female mice treated for 3 months with either a blank or DHT containing capsule; Scale bars =200 μm. (B) Representative images of AR labelling in the rostral arcuate nucleus (rARN), middle arcuate nucleus (mARN), and caudal arcuate nucleus (cARN) in females treated for 3 months with either a blank or DHT containing capsule; Scale bars =200 μm. (C) Mean ± SEM number of AR immunoreactive cells quantified in each hypothalamic area. *n* = 4–7 per group, unpaired Students *t* test, ****p < .0001

**FIGURE 6:**
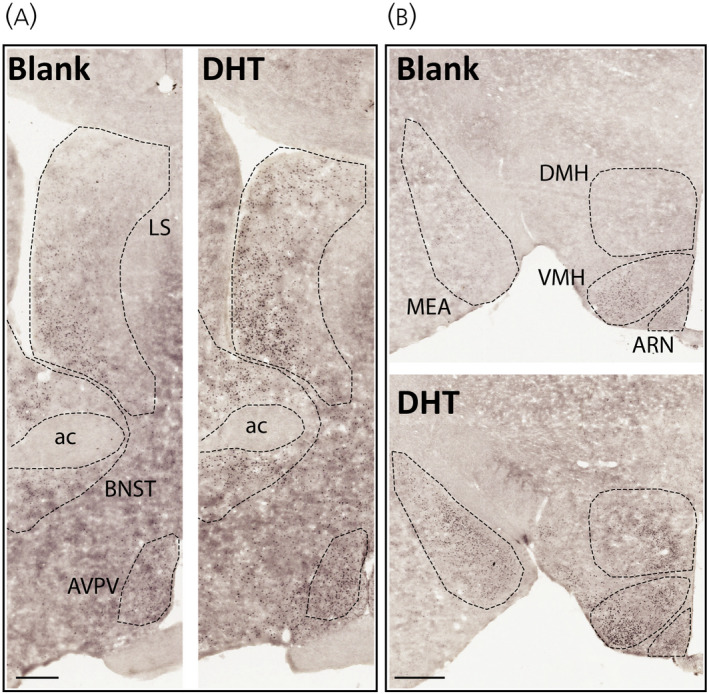
Representative low magnification images of androgen receptor (AR) immunolabelling in anterior (A) and posterior (B) hypothalamic coronal sections. Scale bar in (A) = 250 μm; scale bar in (B) = 400 μm. Ac, anterior commissure; AVPV, anteroventricular periventricular nucleus; LS, lateral septum; BNST, bed nucleus of the stria terminalis; ARN, arcuate nucleus; VMH, venteromedial nucleus of the hypothalmus; DMH, dorsomedial nucleus of the hypothalamus; MEA, medial amygdala

To determine whether chronic androgen excess alters the expression of steroid hormone receptors in the hypothalamus, the number of AR and ERα immunoreactive cells was quantified in hypothalamic nuclei implicated in GnRH neuronal network steroid hormone feedback, including the RP3V and the ARN. ERα positive cells were detected throughout the hypothalamus in the AVPV, PeN, and the rostral to caudal extent of the ARN and no statistically significant changes in the number of ERα positive cells were detected in any region after 3 months of chronic DHT exposure (Figure 4 and Table 1). By contrast, the number of AR positive cells was significantly elevated in all regions examined from mice chronically exposed to DHT (*p* < .0001) (Figure 5 and Table 1). Figure 6 shows lower magnification views of DHT labelling at a more anterior and more posterior level of the hypothalamus to demonstrate the general upregulation of AR expression. Semi‐quantitative analysis of AR and ERα positive cells throughout hypothalamic and some limbic regions shows that, although ERα remains unaltered, AR expression is upregulated in nearly every nuclei in which it is found (Table 2).

**TABLE 1:** Mean ± SEM of androgen receptor (AR) and oestrogen receptor α (ERα) positive neurons throughout the hypothalamus

Region	Receptor	Blank (mean ± SEM)	DHT (mean ± SEM)	*t* statistic, *p* value
AVPV	AR	112.1 ± 32.0	468 ± 26.8	8.288; < .0001
	ERα	252.4 ± 9.8	284 ± 33.5	0.8551; .4107
PeN	AR	27.9 ± 6.6	132 ± 4.3	13.82; < .0001
	ERα	65.1 ± 5.8	73 ± 4.4	0.9921; .3425
rARN	AR	70.1 ± 17.1	270.6 ± 21.5	6.317; < .0001
	ERα	119.3 ± 12.0	129.8 ± 12.9	0.5913; .5663
mARN	AR	65.1 ± 20.6	294.6 ± 22.9	6.667; < .0001
	ERα	207 ± 23.3	212.9 ± 22.1	0.1846; .8569
cARN	AR	65.9 ± 21.4	369.1 ± 18.9	10.15; < .0001
	ERα	234.1 ± 23.1	217.8 ± 15.0	0.6102; .541

Note

AVPV, anteroventral periventricular nucleus; PeN, periventricular nucleus; rARN, rostral arcuate nucleus; mARN, middle arcuate nucleus; cARN, caudal arcuate nucleus. For AR analyses: Blank, *n* = 4 and dihydrotestosterone (DHT), *n* = 7; for ERα analyses: Blank, *n* = 6 and DHT, *n* = 7.

**TABLE 2:** The relative level of androgen receptor (AR) or oestrogen receptor α (ERα) immunoreactive cells across brain regions

Brain region	Relative level of AR+ cells		Relative level of ERα+ cells	
	Blank	DHT	Blank	DHT
Septal nucleus	+	+++	+	+
Bed nucleus of the stria terminalis	+	+++	++	++
Preoptic area	+	+	+	+
Ventromedial preoptic nucleus	++	+++	+	+
Organum vasculosum of the stria terminalis	++	+++	+	+
Medial preoptic nucleus	++	+++	+++	+++
Anteroventral periventricular nucleus	++	+++	+++	+++
Periventricular nucleus	++	+++	++	++
Paraventricular nucleus	+	++	+	+
Lateroanterior hypothalamic nucleus	+	++	−	−
Anterior hypothalamic area	+	++	+	+
Ventromedial hypothalamic nucleus	++	+++	+++	+++
Supraoptic nucleus	+	++	−	−
Suprachiasmatic nucleus	++	+++	−	−
Accessory groups of the supraoptic nucleus	+	+	−	−
Arcuate nucleus	++	+++	+++	+++
Dorsomedial hypothalamic nucleus	+	++	+	+
Lateral hypothalamic area	−	+	−	−
Posterior hypothalamic area	−	+	−	−
Premammillary nucleus	++	+++	−	−
Tuberal nucleus	++	+++	−	−
Zona incerta	−	+	−	−
Paraventricular thalamic nucleus	+	++	−	−
Medial amygdala	++	+++	++	++

Note

−, absence of staining.

+, presence of a few positively labelled cells.

++, presence of many positively stained cells with enough to clearly demarcate specific nuclei.

+++, robust expression of positively labelled cells.

DHT, dihydrotestosterone.

## 4 DISCUSSION

We show that females chronically exposed to the non‐aromatisable androgen DHT from a peri‐pubertal timepoint for 3 months develop the PCOS‐like traits of impaired oestrous cyclicity and increased body weight, as shown previously.^13^, ^15^, ^36^, ^37^, ^38^ Although this mouse model incorporates some of the reproductive and metabolic features typical of clinical PCOS, serial tail‐tip blood sampling identified that the LH pulse frequency in this model is no different from that in healthy control mice. Likewise, chronic DHT exposure from 3 weeks of age had no significant impact on GnRH neuron spine density or on putative GABAergic inputs to GnRH neurons. Chronic DHT exposure significantly upregulated AR expression in several hypothalamic nuclei but had no significant effect on ERα expression. These data suggest that, although DHT exposure from 3 weeks of age models the hyperandrogenism, anovulation and obesity of a PCOS‐like phenotype, it lacks the LH hypersecretion common to clinical PCOS, suggesting that exposure to androgen excess during different developmental windows can drive distinct PCOS‐like phenotypes.

Although the hyperactive pulsatile LH secretion that is typical of clinical PCOS^4^, ^5^ and early androgen exposure models^9^ is not evident in the chronic DHT mouse model, the pathogenesis of PCOS‐like traits in this model are likely to still have some neuronal origin.^15^ DHT‐treated mice with a neuron specific deletion of AR exhibit partially rescued ovulatory function, protection from adipocyte hypertrophy and ameliorated dyslipidemia.^15^ Of interest, knockout of AR from both neurons and adipose tissue protects DHT‐treated mice to an even greater extent, with the majority of mice resuming regular oestrous cyclicity.^39^ The present data suggest that, although chronic DHT exposure initiated from 3 weeks of age in mice is likely acting in the brain given the robust increase in the number of AR‐expressing cells in the hypothalamus, it has no significant effect on LH pulse frequency.

Androgen excess is associated with an impaired ability for gonadal steroid hormones, particularly progesterone, to exert negative feedback effects on GnRH/LH pulse generation. In healthy women, oestradiol upregulates the expression of progesterone receptors (PR) in the hypothalamus and progesterone, in turn, slows GnRH/LH pulse generation in the luteal phase.^40^, ^41^ In women with PCOS, a higher concentration of oestradiol and progesterone are required to lower LH pulse frequency to the same extent as in healthy control women.^42^, ^43^ Androgen excess is thought to mediate this impaired negative feedback, as long‐term AR antagonist treatment of women with PCOS augments the negative feedback effects of oestradiol and progesterone.^44^ Studies in rodent animal models have demonstrated that DHT interferes with the ability of progesterone to reduce the activity of GnRH neurons,^24^ through a mechanism involving GABA neurons. Acute testosterone administration reduces PR mRNA in the hypothalamus and prevents oestradiol‐induced PR mRNA expression,^45^ suggesting one potential mechanism by which androgens may interfere with negative feedback control of the HPG axis. Exposure to androgen excess for a brief period in late gestation develops hyperandrogenic females that exhibit impaired steroid hormone feedback, reduced hypothalamic PR expression and elevated LH pulse frequency,^12^, ^20^ although this does not appear to be the case when androgen excess is introduced later in development.

Chronically elevated androgens in mice may instead generate an “androgen clamp” that interferes with the preovulatory surge and inhibits LH secretion, as has been shown in rats.^45^, ^46^ Acute exposure of female rats to elevated testosterone abolishes the endogenous and the oestrogen‐induced LH surge.^45^ Similarly, chronic DHT exposure in rats from 3 weeks of age abolishes the oestrogen‐primed surge.^46^ By contrast to the present study, however, LH pulses were not detectable in ovariectomised DHT‐treated treated rats.^46^ In a more recent study, LH pulses were measured in serial tail‐tip blood samples from ovariectomised mice with DHT capsules inserted at 5 weeks of age. DHT was found to dose‐dependently reduce LH pulse frequency and amplitude and increase the inter‐pulse interval.^25^ DHT delivery to castrate males and females has been shown to reduce arcuate nucleus kisspeptin cell number and gene expression, supporting a role for AR‐mediated slowing of the GnRH pulse generator.^25^, ^47^ In the present study, DHT had no discernible effect on the pulsatile release of LH in gonadally‐intact female mice. This suggests that the negative feedback impact of DHT may only be measurable following a post‐castration elevation of LH or when delivered at a later timepoint. Unaltered LH pulse frequency in this chronic DHT model suggests that the main impact of androgen actions in the brain may be in interfering with the preovulatory surge mechanism.

The lack of evidence for modified GnRH neuron spine density or presynaptic GABAergic inputs suggests that chronically elevating androgens from 3 weeks of age does not drive major plasticity in the GnRH neuronal network. Acute androgens increase the spine density of hippocampal neurons^48^ and GnRH neuron spine density is increased in PNA models of PCOS.^49^ Although it remains unclear whether spine plasticity in GnRH neurons is associated with changes in synaptic inputs, elevated spine density is associated with physiological periods of elevated neuronal activity.^20^, ^50^ Given the postulated role of GABA neurotransmission in mediating hyperactive GnRH secretion in the PCOS condition,^5^, ^16^, ^51^ the absence of changes in putative GABAergic inputs to the GnRH neuron is aligned with the absence of elevated LH secretion in the chronic DHT model.

Although acute prenatal androgen excess is reported to result in a small but significant increase in ERα and AR expression in the RP3V of adult females,^15^ we identified that chronic DHT dramatically increases AR expression throughout the RP3V and ARN and has no effect on ERα expression in any region investigated. Although ER expression is relatively stable in the brain, there is evidence to suggest that elevated oestradiol down‐regulates ERs^52^ and that ERs are upregulated in the absence of circulating oestradiol.^53^ The absence of ERα expression changes here is in line with the absence of differences in circulating oestradiol in this model.^13^ AR expression in the hypothalamus is well known to be autoregulated by androgens in both males and females.^53^ Here, the number of AR immunoreactive cells in the brain was increased by 4–6‐fold in females chronically exposed to DHT. Although the phenotypes of those neurons with upregulated AR remain to be determined, increased AR expression in the PNA model has been identified in agouti‐related protein neurons,^54^ as well as in ARN kisspeptin neurons.^55^ This robust upregulation of AR supports direct androgen excess actions in the brain; however, the specific circuits and mechanisms involved remain to be determined. A limitation of the present study is the absence of data for PR expression in similar brain areas. The ability to address this was hindered by the limited availability of tissue and reagents, particularly commercially available PR antibodies that work well in mouse brain tissue. Given the absence of evidence for impaired steroid hormone feedback in the chronic androgen model, we would not predict that basal PR expression patterns would be different; however, this remains to be determined.

These data suggest that critical windows exist for AR‐mediated actions that develop PCOS‐like features. Prenatal androgen exposure in preclinical models drives a lean phenotype with elevated LH secretion, whereas exposure that only begins later in life drives an obese phenotype lacking elevated LH secretion. Although elevated LH levels are evident in both lean and obese PCOS patients, LH is more frequently elevated in lean patients and are not consistently elevated in obese PCOS patients.^56^, ^57^ Notably, obesity alone is associated with reduced LH and obese women with PCOS have a reduced LH amplitude compared to their lean counterparts.^58^, ^59^ Androgen actions introduced in later postnatal development or adulthood may have a primary impact on metabolic parameters, including the development of hyperinsulinaemia, which may then contribute to reproductive dysfunction. Challenging this notion, administration of testosterone as part of a gender affirming hormone therapy to female‐to‐male transgender individuals does not promote insulin resistance or a metabolic phenotype.^60^ Additionally, genetic causes of extreme insulin resistance in women result in high circulating testosterone, suggesting that hyperinsulinaemia promotes hyperandrogenism, not necessarily the reverse.^61^ In any case, female hyperandrogenism is associated with both blunting negative feedback control of the HPG axis, as well as clamping down HPG axis output. The outcome may be dependent upon the developmental window of first exposure, the relative level of the androgen exposure, and the aromatisable nature of the elevated androgen. Although artificially increasing DHT enables the specific investigation of AR‐mediated effects, endogenous or exogenous testosterone will result in both ER‐ and AR‐mediated effects following the metabolism of testosterone to oestradiol in the brain.

Together with previous reports on this model, our findings suggest that chronic DHT exposure from 3 weeks of age recapitulates an obese, acyclic, hyperandrogenic phenotype that is not associated with a hyperactive HPG axis. Although there are no perfect models of PCOS, which only naturally occurs in humans and non‐human primates,^9^ preclinical models remain critical with respect to determining the pathogenesis of androgen excess in driving PCOS features in females. Reductionist models such as this that highlight the consequences of female androgen excess can identify novel targets for intervention in a disease that is currently only treated symptomatically as a result of a lack of understanding of its aetiology and pathogenesis.

## ACKNOWLEDGMENTS

Open access publishing facilitated by University of Otago, as part of the Wiley ‐ University of Otago agreement via the Council of Australian University Librarians.

## CONFLICT OF INTERESTS

The authors declare that they have no conflicts of interest.

## AUTHOR CONTRIBUTIONS

**Chris S. Coyle:** Data curation; Formal analysis; Writing – original draft. **Melanie Prescott:** Data curation; Methodology; Supervision. **David J. Handelsman:** Methodology; Resources; Writing – review & editing. **Kirsty A. Walters:** Conceptualization; Project administration; Resources; Writing – review & editing. **Rebecca E. Campbell:** Conceptualization; Funding acquisition; Project administration; Supervision; Writing – original draft; Writing – review & editing.

### PEER REVIEW

The peer review history for this article is available at https://publons.com/publon/10.1111/jne.13110.

## Data Availability

The data that support the findings of the present study are available from the corresponding author upon reasonable request.
